# Supplementary material for: A ‘Spicy’ Mechanotransduction Switch: Capsaicin-Activated TRPV1 Receptor Modulates Osteosarcoma Cell Behavior and Drug Sensitivity
Source: Int J Mol Sci. 2025 Sep 10;26(18):8816. doi: 10.3390/ijms26188816 (PMC12469300; doi:10.3390/ijms26188816)

Supplementary Materials and Methods

Experimental design for investigating the role of MS channels in osteosarcoma mechanosensing

To investigate whether inhibition of a single mechanosensitive (MS) ion channel is sufficient to reproduce the broad inhibitory effects previously observed with GsMTx4—a general MS channel blocker[1] -we focused on TRPV1, a well-characterized member of the MS channel family. Osteosarcoma (OS) cells were subjected to cyclic uniaxial mechanical stretch in the presence or absence of AMG9810, a selective TRPV1 antagonist.

As illustrated in **supplementary Figure S1**, two complementary experimental strategies were employed to assess both immediate and lasting effects of mechanical stimulation:

1. **Immediate-response analysis**:

   Following the 24-hour stretching protocol, cells were analyzed directly on the silicone membrane using immunofluorescence, confocal microscopy, atomic force AFM microscopy and western blotting. These techniques allowed for the assessment of acute morphological and molecular responses to mechanical stretch, including cell orientation the capacity of cells to align along the stretch axis, changes in cytoskeletal organization, alterations in cell boundary architecture and modulation of mechanotransduction signaling markers.

2. **Lasting-response analysis (preconditioned cells)**:

   To assess the longer-term phenotypic consequences of mechanical preconditioning, cells were subjected to a series of functional assays designed to probe their **metastatic potential**:

   - **Adhesion assays and detachment assays** to evaluate cell adherence capacity and stability of attachment to the substrate.
   - **Wound healing** to measure cell migratory capability.
   - **Chemotherapy response assays** using cisplatin and doxorubicin—standard agents in MAP therapy—to determine whether cyclic stretch influences OS **chemosensitivity**.

By comparing these outcomes across stretched and unstretched conditions, with or without TRPV1 inhibition, we aimed to delineate the specific contribution of TRPV1 to the mechanosensitive phenotype of OS cells. These experiments also assessed whether selective TRPV1 inhibition can recapitulate the broader suppressive effects seen with general MS channel blockade.

To evaluate whether nanomolar capsaicin treatment could reproduce mechanical phenotypes and modulate chemoresponsiveness in osteosarcoma (OS) cells, all experimental approaches described in **Supplementary Figure S1**—excluding the cell reorientation analysis—were repeated on both OS cell subtypes. In this case, capsaicin, a chemical activator of TRPV1, was used in the absence of any mechanical stimulation.

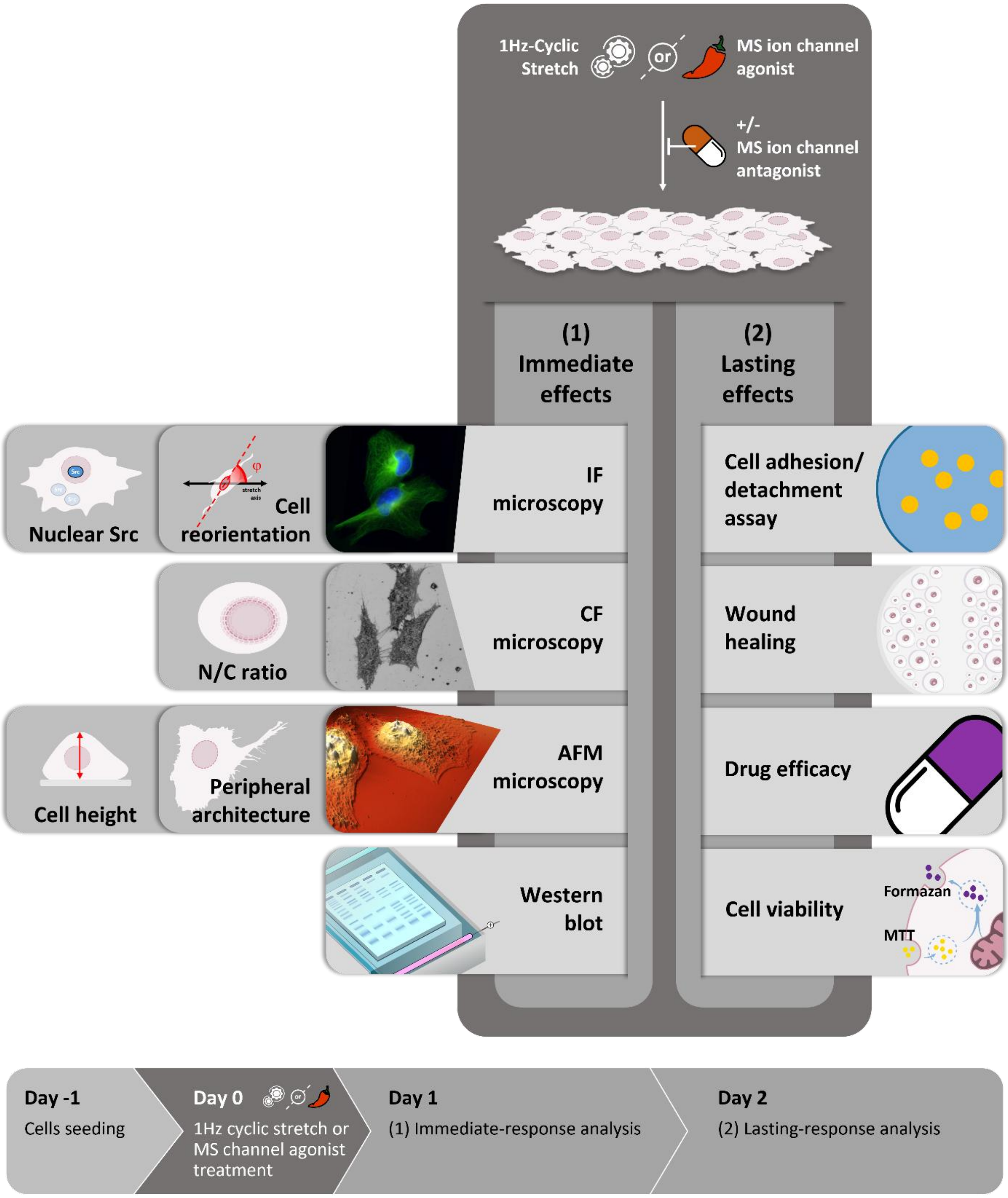

Supplementary Figure S1: Schematic Overview of the Experimental Design and Analytical Workflow. This figure outlines the experimental strategies used to investigate how osteosarcoma (OS) cells reprogram normal mechanosensing, contributing to their aggressive behavior. Two complementary experimental approaches were employed: 1) Immediate Effects—assessed one day after the initial mechanical stretch; 2) Lasting Effects—evaluated on day two to examine the sustained impact of mechanical stimulation.

Detailed procedure for assessing cell reorientation via nuclear immunofluorescence

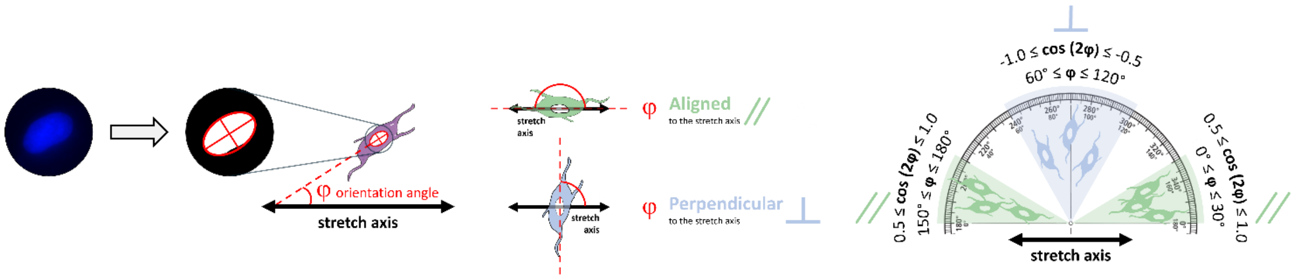

Supplementary figure S2: Schematic representation of nuclear orientation analysis. The image shows a Hoechst-stained nucleus before and after binary conversion. The orientation angle, φ, was measured between the major axis of the fitted ellipse and the direction of stretch. Nuclei were classified as perpendicular when φ ranged from 60° to 120° ($60° \leq \varphi \leq 120°$), parallel when φ was between 0° and 30° or 150° and 180° ($0° \leq \varphi \leq 30°$ or $150° \leq \varphi \leq 180°$), and randomly oriented when φ fell between 30° and 60° or 120° and 150° ($30° \leq \varphi \leq 60°$ or $120° \leq \varphi \leq 150°$). For simplified analysis, φ was transformed into the orientation parameter cos(2φ). Values between -0.5 and 0.5 indicate random orientation ($-0.5 \leq \cos(2\varphi) \leq 0.5$), values between 0.5 and 1 indicate parallel orientation ($0.5 \leq \cos(2\varphi) \leq 1$), and values between -1 and -0.5 indicate perpendicular orientation ($-1 \leq \cos(2\varphi) \leq -0.5$) relative to the stretch axis.

Capsaicin and AMG9810 as a potential mechanomimetic tools for modulation of cell behavior and drug sensitivity in OS cells

While TRPV1 is known to be activated by various endogenous stimuli—including heat, acidic pH, and lipid metabolites [2]- we focused on capsaicin due to its status as a well-characterized, archetypal TRPV1 agonist. As a widely consumed nutraceutical compound, capsaicin is readily absorbed in the human body, making it a biologically relevant tool for studying TRPV1's function in osteosarcoma (OS) cells.

Although capsaicin has an established EC_{50} of ~99 nM for TRPV1 activation [2], it is a pleiotropic compound known to interact with multiple cellular targets, such as HSP90, milk proteins, and human transferrin. Its effects are dose- and cell-type dependent, with both pro- and anti-cancer properties reported in the literature. To specifically investigate TRPV1-mediated responses while minimizing off-target effects, we limited our analysis to low nanomolar concentrations [3], [4].

To pharmacologically inhibit TRPV1, we used AMG9810—a potent aryl cinnamide-class antagonist with IC_{50} values in the low nanomolar range [5] —at a concentration of 560 nM. This dose, approximately 10-fold higher than that of capsaicin, was chosen to ensure effective inhibition of both chemical and mechanical TRPV1 activation.

Our primary objective was to determine whether capsaicin could act as a "mechanomimetic" agent—mimicking the effects of mechanical stimulation on OS cells—without inducing cytotoxicity in healthy osteoblasts. We began by reproducing previously observed mechanically induced changes in OS cell behavior (**Figure 3A–C** and **Supplementary Figure S3**) and by validating the cytotoxic effects of high-dose capsaicin reported in the literature. We then investigated whether low-dose capsaicin could replicate the drug-sensitizing effects seen in mechanically preconditioned OS cells **(Supplementary Figure 3E–F** and **Supplementary Figure S3**).

Given the lack of cytotoxicity at nanomolar concentrations, we next assessed capsaicin's potential as an adjuvant to standard MAP chemotherapy agents, cisplatin and doxorubicin. To further dissect the role of TRPV1 signaling in this context, we examined the effects of TRPV1 inhibition using AMG9810, both alone and in combination with capsaicin.

At nanomolar concnetrations Neither Capsaicin nor AMG, Individually or Combined, Affects Cisplatin-Induced Cytotoxicity in hFOB Cells

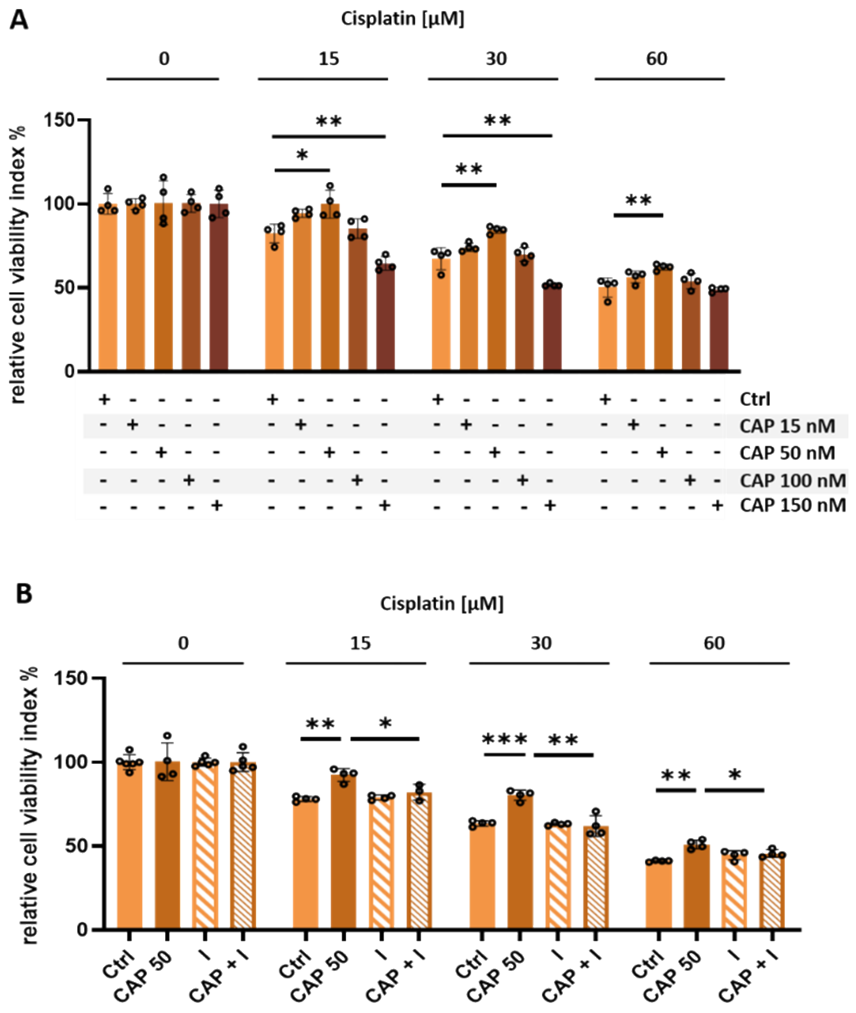

Supplementary Figure S3. Cisplatin cytotoxicity in the presence or absence of capsaicin ,AMG, individually or combination, in hFOB cells. Panel (A) Cell viability of hFOB cells treated with cisplatin following pretreatment with increasing concentrations of capsaicin (0, 15, 50, 100, and 150 nM)**. Panel (B)** Effect of TRPV1 inactivation on cisplatin-induced cytotoxicity in hFOB cells. Conditions include: cisplatin-only control (Ctrl), capsaicin 50 nM (CAP 50), TRPV1 antagonist AMG9810 (560 nM; denoted as "I"), and combined treatment with capsaicin and AMG9810 (CAP + I). All data are derived from the best of three biological replicates, each with at least three technical replicates per condition. Statistical analyses were performed using Student's *t*-test. Statistical significance is denoted as follows: $p < 0.05$ (*), $p < 0.01$ (**), $p < 0.001$ (***), $p < 0.0001$ (****).

Nuclear size in hFOB cells is not affected by MS ion channel agonists ASP7663 and capsaicin

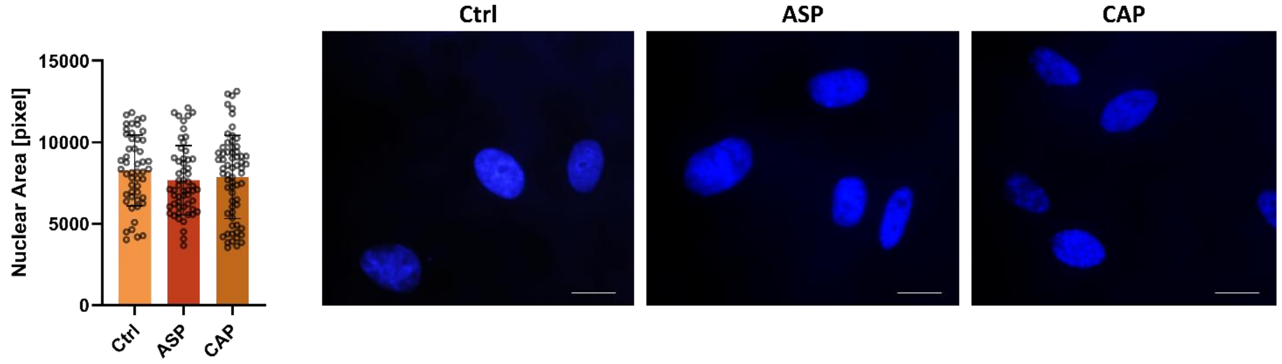

Supplementary Figure S4: Chemical Activation of either TRPV1 or TRPA1 Channels hFOB Cells does not alters nuclear size. Representative fluorescence microscopy images showing the effects of soluble, channel-specific agonists on nuclear morphology. Cells were treated with 50 nM Capsaicin (**CAP**; TRPV1 agonist) or 50 nM ASP7663 (**ASP**; TRPA1 agonist). Nuclei were stained with Hoechst, and images were acquired at 40× magnification; scale bar = 25 µm. Nuclear areas were quantified using the binary thresholding function in ImageJ (v1.52). Data represent three biological replicates, with a minimum of 45 cells per condition.

Nuclear height and peripheral roughness do not significantly change upon TRPV1 activation

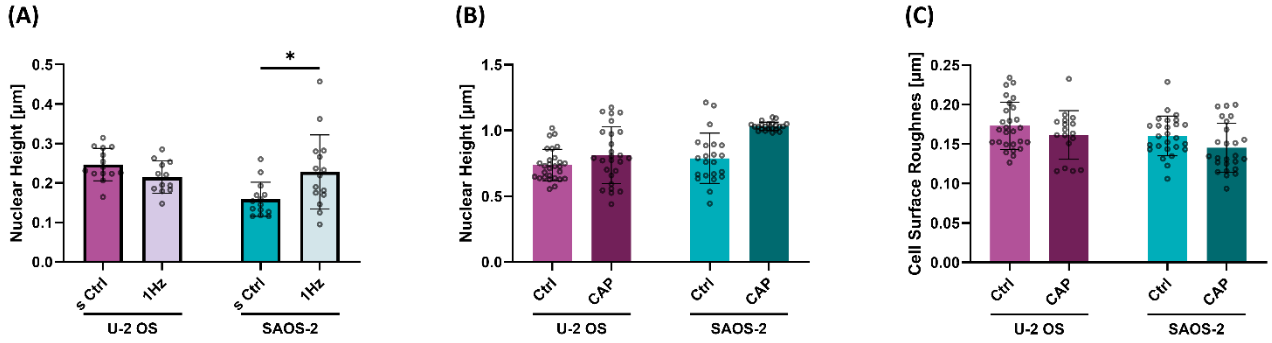

Supplementary Figure S5: **Atomic force microscopy (AFM)-based analysis of U-2 OS and SAOS-2 cells after mechanical or chemical activation of TRPV1 channels. Panels A and B** show measurements of nuclear height. **Panel C** presents quantification of cell surface roughness. Data represent three biological replicates, with a minimum of 12 cells per condition. Statistical analyses were performed using Student's *t*-test. Statistical significance is denoted as follows: $p < 0.05$ (*), $p < 0.01$ (**), $p < 0.001$ (***), $p < 0.0001$ (****).

Methodology for AFM-based quantification of cell edge architecture complexity

Fractals are complex geometric structures characterized by fine detail at arbitrarily small scales [6]. A defining feature of fractals is self-similarity—the repetition of patterns or branching across multiple length scales. In other words, zooming in on a small region of a fractal reveals structural features resembling those of the whole. This intrinsic complexity often results in large surface areas or intricate boundaries, making fractal patterns widespread in biology, where surface-related phenomena play critical roles—such as in cell adhesion, membrane trafficking, signaling receptor distribution, and cytoskeletal remodeling [7]. Unlike standard Euclidean metrics, which are suited for simple geometric shapes (e.g., lines or smooth curves), FD analysis is particularly effective in capturing the complexity of biologically relevant, irregular architectures.

While the dimension of simple geometries (e.g., lines or smooth surfaces) can be intuitively understood as the minimum number of coordinates needed to describe all points, this approach fails when applied to irregular structures like fractals. To quantify the dimensionality of such complex forms, the box-counting dimension—a type of fractal dimension (FD)—is used.

Let S be a subset in a D-dimensional Euclidean space, and let N(ε) be the minimum number of D-dimensional cubes of side length ε needed to cover S. The **box-**counting dimension d_f is then defined as:

$$d_f = \lim_{\boldsymbol{\varepsilon} \to \mathbf{0}} \frac{\ln \mathrm{N}(\boldsymbol{\varepsilon})}{\ln (1/\boldsymbol{\varepsilon})} \qquad \text{eq (1)}$$

When applied to simple shapes, this method yields expected results (e.g., d_f==1 for a line and d_f=2 for a filled square). However, for more complex, one-dimensional structures—such as fractal curves—this calculation yields a dimension between 1 and 2, depending on the intricacy of the contour.

Fractal dimension analysis is a powerful quantitative tool for assessing surface complexity in biological systems. It has been applied, for example, to differentiate between healthy and cancerous cervical epithelial cells by analyzing entire AFM-scanned surfaces [8], and to measure the effect of actin polymerization inhibitors on cell boundary roughness in human epithelial cells [9] .

In the present study, FD analysis was performed on AFM images of U-2 OS and SAOS-2 cells. Each AFM image was processed by cropping the cell outline and applying a contrast filter to facilitate accurate identification of the cell periphery (supplementary **Figure S6A, B**). A one-pixel-wide binary mask of the cell edge was then extracted (supplementary **Figure S6C-D** shows the profile of three lamellipodia-like protrusions, each approximately 100 nm in width—well within the resolution capacity of AFM. This highlights the utility of AFM in resolving fine peripheral features that conventional light microscopy may fail to detect.

The fractal dimension of each cell's boundary was then computed by applying Formula 1 to the extracted edge masks using the box-counting method, as illustrated in supplementary **Figure S6E**. This approach allowed for a quantitative comparison of cell edge complexity under different treatment conditions.

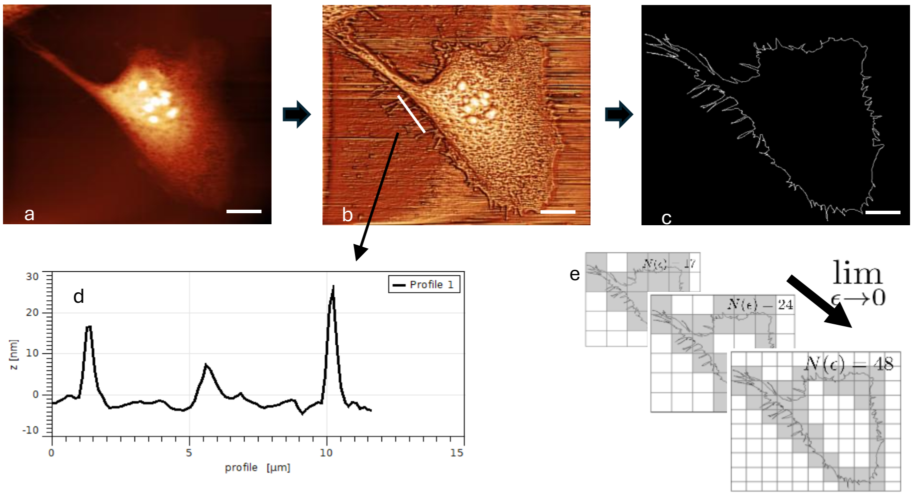

Supplementary Figure S6: **Fractal analysis procedure**. a) Original AFM image of representative U-2 OS cells; b) contrast enhancement and profile along 3 fingers; c) extracted black and white pixel mask; d) profile of three fingers showing width of approximately 100 nm and e) illustration of the box counting method. For a given dimension of ε the total number of boxes containing at least on pixel of the outline were counted to give N(ε**).**

Semi-automated quantification of nuclear Src levels using CellProfiler pipeline

Fluorescence images were analyzed using a customized CellProfiler 4.2.8 pipeline adapted from ImageJ-based workflows. Six datasets, each with three experimental conditions, were processed, analyzing at least seven paired nucleus and c-Src images per condition. The same pipeline was applied to all datasets, with only minimal adjustments for optimal object detection, as detailed in **Supplementary Table S1**.

Images were converted to grayscale, background noise was corrected, and signals for nuclei and c-Src were enhanced based on an updated image processing method. Segmentation allowed identification of nuclear and c-Src regions, as well as a peripheral background region. Morphological and fluorescence intensity data were extracted, and nuclear c-Src levels were quantified by calculating the Corrected Total Cell Fluorescence (CTCF) in Microsoft Excel.

The CellProfiler pipeline used will be publicly available upon publication.

CTCF was calculated using the following formula:

$$CTCF = Nuclear\ cSrc\ Integrated\ Density - (Background\ Mean\ Intensity \times Nuclear\ Area)$$

Where:

$$Nuclear\ cSrc\ Integrated\ Density = cSrc\ Mean\ Intensity \times Nuclear\ Area$$

CTCF values were visualized and analyzed using GraphPad Prism (v10.4, San Diego, CA, USA).

Supplementary Table S1

CLUSTER	MODULE NAME	SETTINGS
**Converting image to grayscale**<br>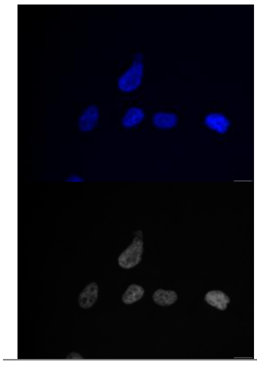	ColorToGray	Select the input image: Nuclei_RGB<br>Conversion method: Combine<br>Name the output image: Nuclei_Gray<br>Image type: RGB<br>Relative weight of the red channel: 1<br>Relative weight of the green channel: 1<br>Relative weight of the blue channel: 1
**Decrease nuclei background signal**<br>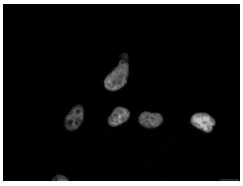	Threshold	Select the input image: Nuclei_Gray<br>Name the output image: Threshold_Nuclei<br>Threshold strategy: Global<br>Thresholding method: Minimum Cross-Entropy<br>Threshold smoothing scale: 0<br>Threshold correction factor: 1<br>Lower and upper bounds on threshold: 0.0 – 1.0<br>Log transform before thresholding: No
	MaskImage	Select the input image: Nuclei_Gray<br>Name the output image: Background_Nuclei<br>Use objects or an image as a mask: Image<br>Select image for mask: Threshold_Nuclei<br>Invert the mask: Yes
	Watershed	Use advanced settings: No<br>Select the input image: Background_Nuclei<br>Name the output object: Background_Nuclei_Area<br>Generate from: Distance<br>Footprint: 8<br>Downsample: 1
	MeasureImage Intensity	Select images to measure: Nuclei_Gray<br>Measure the intensity only from area enclosed by objects: Yes<br>Select input object sets: Background_Nuclei_Area<br>Calculate custom percentiles: No
	ImageMath	Operation: Subtract<br>Name the output image: Nuclei_Background_Subtraction<br>Image or measurement: Image<br>Select the first image: Nuclei_Gray<br>Multiply the first image by: 1<br>Image or measurement: Measurement

		Category: Intensity<br>Measurement: MeanIntensity<br>Image: Nuclei_Gray_Background_Nuclei_Area<br>Multiply the second image by: 1<br>Raise the power of the result by: 1<br>Multiply the result by: 2<br>Add to result: 0<br>Set values less than 0 equal: Yes<br>Set values greater than 1equal to 1? Yes
**Converting image to grayscale**<br>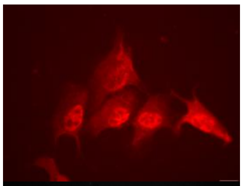<br>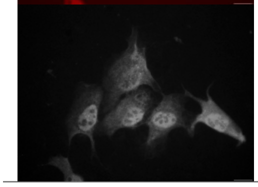	ColorToGray	Select the input image: SRC_RGB<br>Conversion method: Combine<br>Name the output image: SRC_Gray<br>Image type: RGB<br>Relative weight of the red channel: 2<br>Relative weight of the green channel: 1<br>Relative weight of the blue channel: 1
**Decrease Src background signal**<br>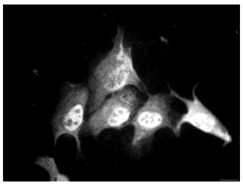	Threshold	Select the input image: SRC_Gray<br>Name the output image: Threshold_SRC<br>Threshold strategy: Adaptive<br>Thresholding method: Otsu<br>Two-class or three-class thresholding: Three classes<br>Assign pixels in the middle intensity class to the foreground or the background: Foreground<br>Threshold smoothing scale: 0<br>Threshold correction factor: 1.1<br>Lower and upper bounds on threshold: 0.0 – 1.0<br>Size of adaptive windows: 50<br>Log transform before thresholding: no
	MaskImage	Select the input image: SRC_Gray<br>Name the output image: Background_SRC<br>Use objects or an image as a mask: Image<br>Select image for mask: Threshold_SRC<br>Invert the mask: Yes
	Watershed	Use advanced settings: No<br>Select the input image: Background_SRC<br>Name the output object: Background_SRC_Area<br>Generate from: Distance<br>Footprint: 8<br>Downsample: 1
	MeasureImage Intensity	Select images to measure: SRC_Gray

		Measure the intensity only from area enclosed by objects: Yes<br>Select input object sets: Background_SRC_Area<br>Calculate custom percentiles: No
	ImageMath	Operation: Subtract<br>Name the output image: SRC_Background_Subtraction<br>Image or measurement: Image<br>Select the first image: SRC_Gray<br>Multiply the first image by: 1<br>Image or measurement: Measurement<br>Category: Intensity<br>Measurement: MeanIntensity<br>Image: SRC_Gray_Background_SRC_Area<br>Multiply the second image by: 1<br>Raise the power of the result by: 1<br>Multiply the result by: 2<br>Add to result: 0<br>Set values less than 0 equal: Yes<br>Set values greater than 1 equal to 1: Yes
**Objects Identification**		
**Nuclei**<br>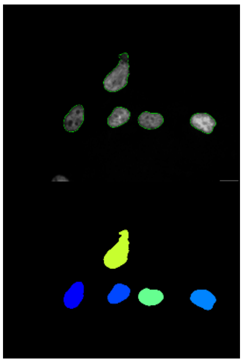	IdentifyPrimary Objects	Use advanced settings: yes<br>Select the input image: Nuclei_Background_Subtraction<br>Name the primary object to be identified: Nuclei_Obj<br>Typical diameter of objects in pixel units: 70-170<br>Discard objects outside the diameter range: yes<br>Discard objects touching the border of the image: yes<br>Threshold strategy: Adaptive<br>Thresholding method: Minimum Cross-Entropy<br>Threshold smoothing scale: 0<br>Threshold correction factor: 1.1<br>Lower and upper bounds on Treshold: 0.0-1.0<br>Log transform before thresholding: no<br>Method to distinguish clumped objects: Shape<br>Method to draw dividing lines between clumped objects: Shape<br>Automatically calculate size of smoothing filter for declumping: No<br>Size of smoothing filter: 5<br>Automatically calculate minimum allowed distance between local maxima: Yes<br>Speed up by using lower-resolution image to find local maxima: Yes<br>Display accepted local maxima: No<br>Fill holes in identified objects: after both thresholding

		and declumping<br>Handling of objects if excessive number of objects is identified: Continue
**Src region**<br>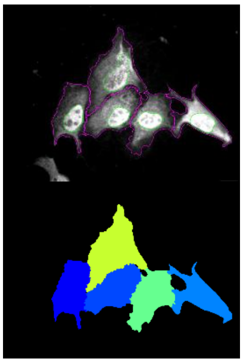	IdentifySecondaryObjects	Select the input image: SRC_Background_Subtraction<br>Select the input objects: Nuclei_Obj<br>Name the object to be identified: SRC_Fluorescent_Region<br>Select the method to identify the secondary objects: Watershed - Image<br>Threshold strategy: Global<br>Thresholding method: Otsu<br>Two-class or three-class thresholding: Three classes<br>Threshold smoothing scale: 5<br>Threshold correction factor: 0.4<br>Lower and upper bounds on Threshold: 0.0-1.0<br>Log transform before thresholding: No<br>Fill holes in identified objects: Yes<br>Discard secondary objects touching the border of the image: No
**Single-cell peripheral background region**<br>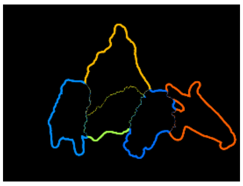	ExpandOrShrinkObjects	Select the input objects: SRC_Fluorescent_Region<br>Name the output objects: Expand_SRCFluRe_Max<br>Select the operation: Expand objects by a specified number of pixels<br>Number of pixels by which to expand or shrink: 20
	ExpandOrShrinkObjects	Select the input objects: SRC_Fluorescent_Region<br>Name the output objects: Expand_SRCFluRe_Min<br>Select the operation: Expand objects by a specified number of pixels<br>Number of pixels by which to expand or shrink: 20
	IdentifyTertiaryObjects	Select the larger identified objects: Expand_SRCFluRe_Max<br>Select the smaller identified objects: Expand_SRCFluRe_Min<br>Name the tertiary objects to be identified: Cells_Background<br>Shrink the smaller object prior to subtraction: Yes
**Overlay the identified regions on the raw image**<br>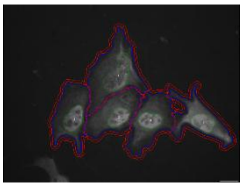	OverlayOutlines	Display outlines on a blank image: No<br>Select image on which to display outlines: SRC_Gray<br>Name the output image: Cells_Nu_SRCFluRe_Back_Outlines<br>Outline display mode: Color<br>How to outline: Inner<br>Select objects to display: Cells_Background<br>Select outline color: Red (R 255, G 000, B 000)<br>Select objects to display: Nuclei_Obj<br>Select outline color: Green (R 000, G 255, B 000)<br>Select objects to display: SRC_Fluorescent_Region

		Select outline color: Blue (R 000, G 000, B 255)
	DisplayData OnImage	Display object or image measurements: Object<br>Select the input objects: Nuclei_Obj<br>Measurement to display, Category: Number<br>Measurement to display, Measurement: Object_Number<br>Display background image: Yes<br>Select the image on which to display the measurements: Cells_Nu_SRCFluRe_Back_Outlines<br>Display mode: Text<br>Font: Calibri, normal, 12<br>Use scientific notation: No<br>Name the output image that has the measurements displayed: DispalyImage<br>Image elements to save: Image
	DisplayData OnImage	Display object or image measurements: Image<br>Measurement to display, Category: FileName<br>Measurement to display, Measurement: SRC_RGB<br>Display background image: Yes<br>Select the image on which to display the measurements: DisplayImage<br>Display mode: Text<br>Font: Calibri, normal, 10<br>Use scientific notation: No<br>Name the output image that has the measurements displayed: DisplayImage_FileName<br>Image elements to save: Image
**Measurement**	MeasureObject SizeShape	Select object sets to measure: Nuclei_Obj<br>Calculate the Zernike features: No<br>Calculate the advanced features: No
	MeasureObject Intensity	Select images to measure: SRC_Gray<br>Select the objects to measure: Cells_Background; Nuclei_Obj
**Export data**	ExportTo Spreadsheet	Overwrite existing files without warning: No<br>Add image metadata columns to your object data file: No<br>Add image file and folder names to your object data file: No<br>Representation of Nan/Inf: NaN<br>Select the measurements to export: No<br>Calculate the per-image mean values for the object measurements: No<br>Calculate the per-image median values for the object measurements: No<br>Calculate the per-image standard deviation values for object measurements: No

		Create a GenePattern GCT file: No<br>Export all measurement types: Yes
	SaveImages	Select the type of image to save: Image<br>Select the image to save: DisplayImage_FileName<br>Select method for constructing file names: Sequential numbers<br>Enter file prefix:_CTCF_DispalyedAnalysis<br>Saved file format: tiff<br>Image bit depth: 8-bit integer<br>Save with lossless compression: Yes<br>Output file location: Same folder as image

References

[1] A. Buglione *et al.*, 'GsMTx-4 venom toxin antagonizes biophysical modulation of metastatic traits in human osteosarcoma cells', *Eur J Cell Biol*, vol. 104, no. 1, p. 151469, Mar. 2025, doi: 10.1016/j.ejcb.2024.151469.

[2] M. F. McCarty, J. J. DiNicolantonio, and J. H. O'Keefe, 'Capsaicin may have important potential for promoting vascular and metabolic health', *Open Heart*, vol. 2, no. 1, p. e000262, 2015, doi: 10.1136/openhrt-2015-000262.

[3] F. Yang *et al.*, 'Structural mechanism underlying capsaicin binding and activation of the TRPV1 ion channel', *Nat Chem Biol*, vol. 11, no. 7, pp. 518–524, Jul. 2015, doi: 10.1038/nchembio.1835.

[4] H. I. Ingólfsson *et al.*, 'Phytochemicals perturb membranes and promiscuously alter protein function', *ACS Chem Biol*, vol. 9, no. 8, pp. 1788–1798, Aug. 2014, doi: 10.1021/cb500086e.

[5] N. R. Gavva *et al.*, 'AMG 9810 [(E)-3-(4-t-butylphenyl)-N-(2,3-dihydrobenzo[b][1,4] dioxin-6-yl)acrylamide], a novel vanilloid receptor 1 (TRPV1) antagonist with antihyperalgesic properties', *J Pharmacol Exp Ther*, vol. 313, no. 1, pp. 474–484, Apr. 2005, doi: 10.1124/jpet.104.079855.

[6] S. H. Strogatz, *Nonlinear Dynamics and Chaos: With Applications to Physics, Biology, Chemistry, and Engineering*, 3rd ed. Boca Raton: Chapman and Hall/CRC, 2024. doi: 10.1201/9780429398490.

[7] S. Havlin *et al.*, 'Fractals in biology and medicine', *Chaos, Solitons & Fractals*, vol. 6, pp. 171–201, 1995, doi: 10.1016/0960-0779(95)80025-C.

[8] N. V. Guz, M. E. Dokukin, C. D. Woodworth, A. Cardin, and I. Sokolov, 'Towards early detection of cervical cancer: Fractal dimension of AFM images of human cervical epithelial cells at different stages of progression to cancer', *Nanomedicine: Nanotechnology, Biology and Medicine*, vol. 11, no. 7, pp. 1667–1675, Oct. 2015, doi: 10.1016/j.nano.2015.04.012.

[9] Y. J. Kim, H.-D. Kim, H. H. Kim, S.-M. Shin, C. J. Kang, and K. H. Lee, 'Fractal analysis of cell boundary ultrastructure imaged by atomic force microscopy', *Animal Cells and Systems*, vol. 19, no. 3, pp. 161–167, May 2015, doi: 10.1080/19768354.2015.1037347.

Raw images of western blotting analyses

PVDF Membrane filter related to Figure 5A: SRC (60 kDa) and GAPDH (37 kDa) U-2 OS

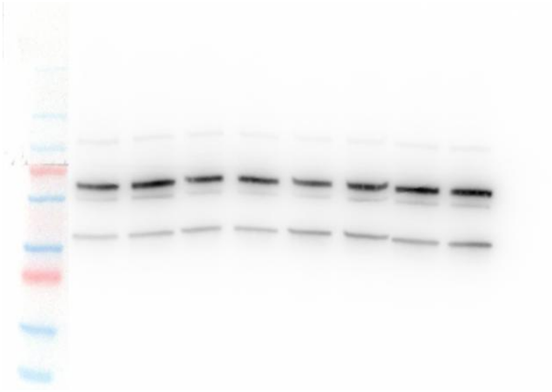

PVDF Membrane filter related to Figure 5A: SRC (60 kDa) and GAPDH (37 kDa) SAOS-2

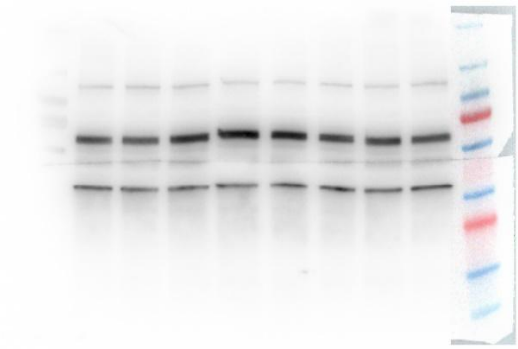

PVDF Membrane filters related to Figure 5A: acetylated histone 3 (17 kDa) and Histone 3 (17 kDa) U-2 OS

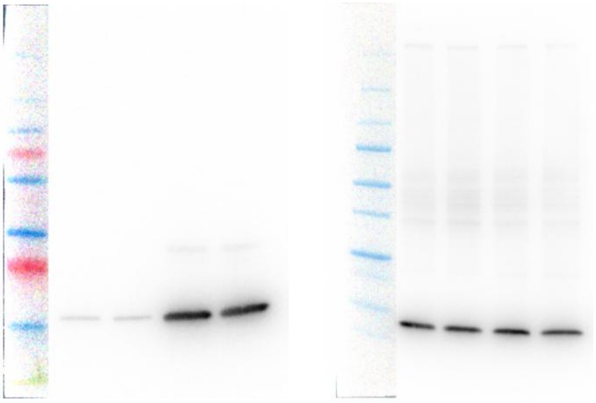

PVDF Membrane filters related to Figure 5A: acetylated histone 3 (17 kDa) and Histone 3 (17 kDa) SAOS-2

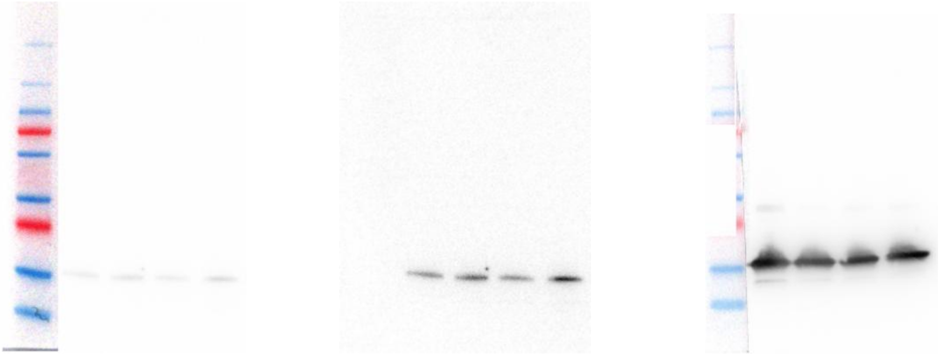

PVDF Membrane filter related to Figure 5B: SRC (60 kDa) and GAPDH (37 kDa) U-2 OS

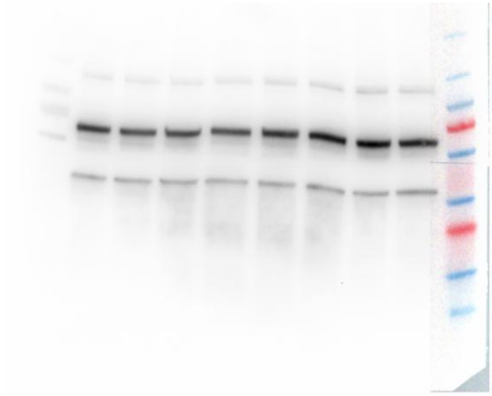

PVDF Membrane filter related to Figure 5B: SRC (60 kDa) and GAPDH (37 kDa) SAOS-2

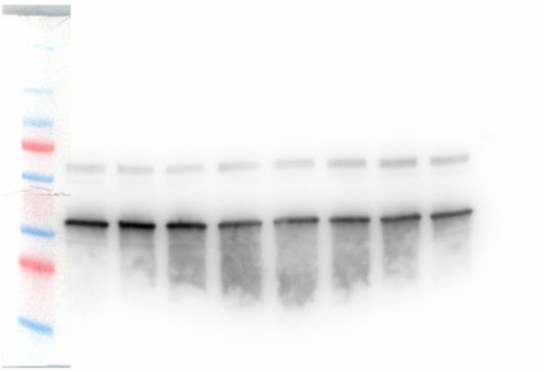

PVDF Membrane filters related to Figure 5B: acetylated histone 3 (17 kDa) and Histone 3 (17 kDa) U-2 OS

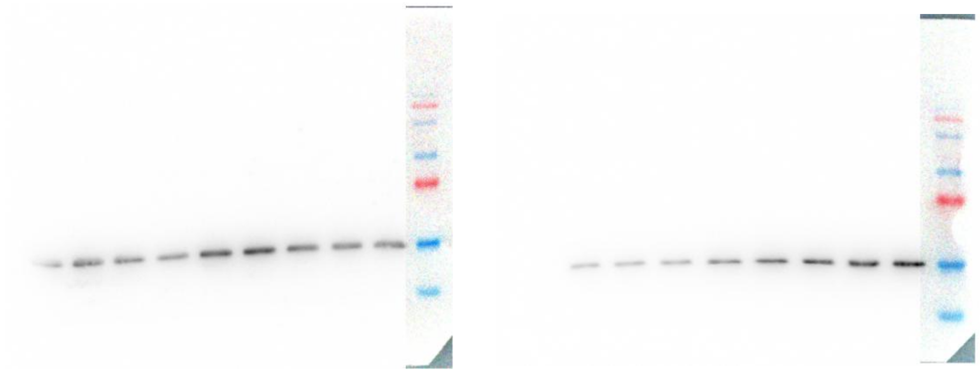

PVDF Membrane filters related to Figure 5B: acetylated histone 3 (17 kDa) and Histone 3 (17 kDa) SAOS-2

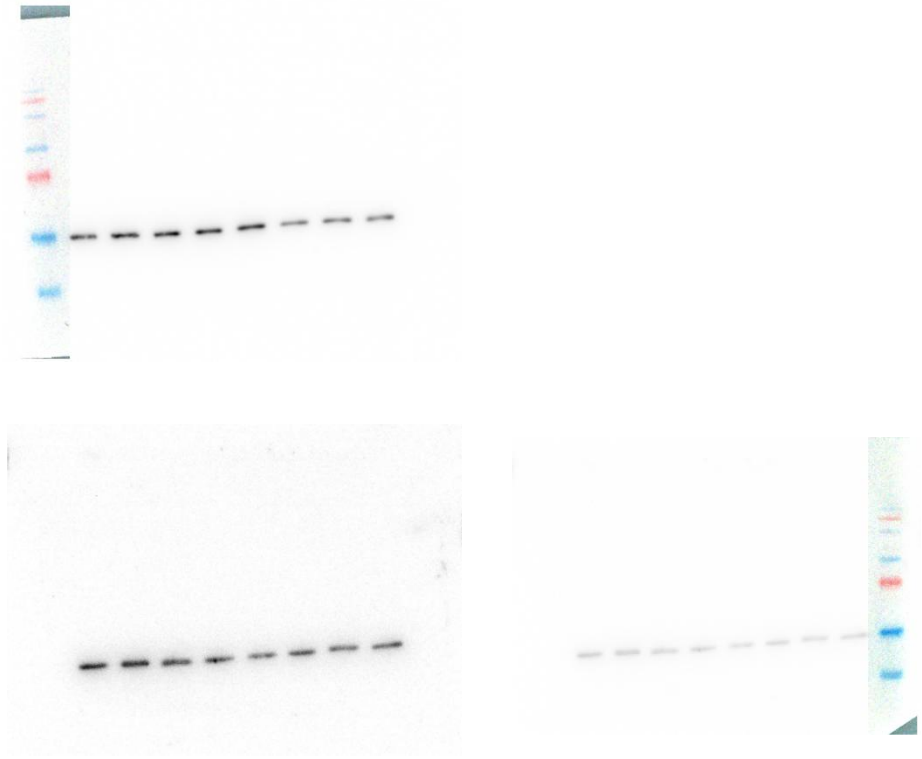

PVDF Membrane filter related to Figure 5E and 5F: SRC (60 kDa) and GAPDH (37 kDa) hFOB

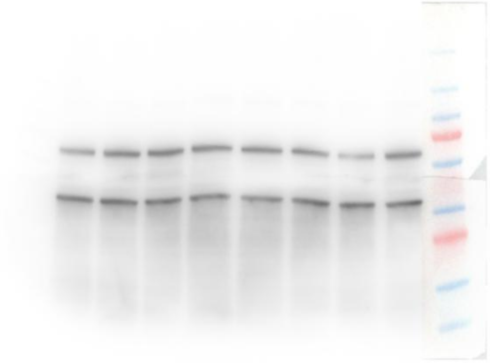

Supplement: Supplementary file 1 [file ijms-26-08816-s001.zip › ijms-3842360-supplementary.pdf]
